# Microalgae-Based 3D Bioprinting: Recent Advances, Applications and Perspectives

**DOI:** 10.3390/md23090342

**Published:** 2025-08-27

**Authors:** Jinhui Tang, Jiahui Sun, Jinyu Cui, Xiangyi Yuan, Guodong Luan, Xuefeng Lu

**Affiliations:** 1 Key Laboratory of Photoelectric Conversion and Utilization of Solar Energy, Qingdao Institute of Bioenergy and Bioprocess Technology, Chinese Academy of Sciences, Qingdao 266101, China; tangjh@qibebt.ac.cn (J.T.); sunjh@qibebt.ac.cn (J.S.); cuijinyu@qibebt.ac.cn (J.C.); yuanxiangyi@qibebt.ac.cn (X.Y.); 2 Shandong Energy Institute, Qingdao 266101, China; 3 Qingdao New Energy Shandong Laboratory, Qingdao 266101, China; 4 College of Life Science, University of Chinese Academy of Sciences, Beijing 100049, China; 5 Laboratory for Marine Biology and Biotechnology, Qingdao National Laboratory for Marine Science and Technology, Qingdao 266101, China

**Keywords:** 3D bioprinting, microalgae, engineered living materials, spatially customized structure

## Abstract

Three-dimensional bioprinting integrating living cells and bioactive materials enables the fabrication of scaffold structures supporting diverse cellular growth and metabolism. Microalgae are among the most promising microbial platforms for the construction of photosynthetic cell factories, while the current industrial-scale cultivation of microalgae remains predominantly dependent on traditional liquid submerged systems, imposing limitations on commercial viability due to both process and economic constraints. Encapsulation of microalgae within bioactive matrices combined with 3D bioprinting to fabricate customized structures has been explored to address the limitations of submerged cultivation, which are expected to expand microalgal applications and establish new research directions in microalgal biotechnology. This review analyzes both matrices and methods of 3D bioprinting, summarizing the advancement of microalgae-based 3D bioprinting into six main domains including living building materials, biophotovoltaics, photosynthetic biosynthesis, bioremediation, tissue engineering, and food engineering. Lastly, synthetic biology-informed perspectives are provided on future developments of 3D bioprinting technologies and their potential in microalgal research.

## 1. Introduction

Three-dimensional (3D) printing, formally known as additive manufacturing (AM), demonstrates a fabrication by constructing objects through layer by layer deposition of printing substrates. This technology transforms computer-aided design (CAD) models into physical entities through a systematic process involving three key steps: digital model segmentation (“slicing”) into printable layers, material deposition guided by sliced data, and sequential layer fusion to form final three-dimensional structures [1]. The advent of biological additive manufacturing has further extended this paradigm, giving rise to 3D bioprinting, an advanced technique that integrates living cells and bioactive components into precisely engineered scaffolds that mimic native tissue architecture and functions [2,3]. Three-dimensional bioprinting has demonstrated advantages in creating tailored cellular microenvironments, combining rapid scaffold fabrication with spatial control over bioactive element distribution [4]. The key to 3D bioprinting technology lies in the development of specialized bioinks, meaning cell-laden biomaterials requiring optimized rheological properties (including shear-thinning behavior and post-printing structural fidelity) to ensure both printability and cellular viability [5,6,7,8]. Advancements in biomaterial science and technology have significantly expanded the repertoire of printable matrices.

Microalgae are a group of photoautotrophic microorganisms encompassing both prokaryotic and eukaryotic species, exhibiting remarkable adaptability across aquatic and terrestrial ecosystems [9,10]. As the essential contributors to global carbon sequestration, microalgae provide approximately 50% of the total primary productivity while maintaining crucial biogeochemical cycles through their carbon fixation capacities [11]. The expanding fields of microalgal biotechnology and bioengineering have permeated multiple industrial sectors, with selected or engineered microalgal strains demonstrating potential in carbon-negative manufacturing, ecological restoration, biomedical innovation, and sustainable agriculture. Advancements in synthetic biotechnology are empowering the reconstruction of the microalgal genetic and metabolic frameworks with greater precision, further broadening the potential applications. By extensively remodeling the metabolism network, carbon flow derived from photosynthesis activities can be directed into the natural or non-natural pathway for the synthesis of diverse metabolites [12]. In the field of biomedicine, microalgae-derived systems show exceptional promise owing to their intrinsic biocompatibility, multifunctional surface architectures, and bioactive metabolite profiles. Emerging applications span contrast-enhanced imaging modalities [13], drug delivery [14], hypoxia-responsive tumor therapeutics [15], and regenerative wound matrices [16], innovations leveraging the photosynthetic machinery and evolutionary tuned stress responses. Despite these advancements, industrial applications of microbial biotechnology and bioengineering are facing critical biological and engineering constraints. Conventional submerged cultivation systems suffer from inherent limitations: light attenuation due to the self-shading restricts biomass accumulation, while downstream processing challenges compromise product recovery efficiency. Furthermore, exposed cultivation induces physiological stress responses that diminish cellular viability and metabolic output [17]. These bottlenecks underscore the urgent need for innovative cultivation paradigms that decouple photosynthetic efficiency from cell density effects while enabling continuous product harvesting.

**Figure 1:**
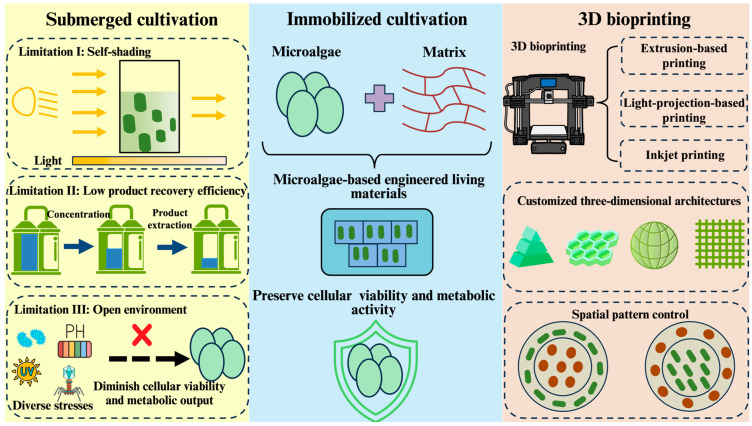
Comparison of two cultivation methods for microalgae and advantages of 3D bioprinting for microalgae. Limitations of submerged cultivation: (I) under high-density cultivation conditions, light is absorbed and scattered by microalgal cells as light penetrates the liquid culture medium. With increasing culture depth and cell density, light intensity exponentially attenuates, impairing photosynthetic efficiency and growth rates of the microalgae [23,24]. (II) In liquid cultivation systems, a significant reduction in overall target metabolite product yield is caused by sequential steps of downstream processing including concentration, separation, and extraction. (III) Under open cultivation conditions, microalgal cells are subjected to multiple stress factors including temperature fluctuations, extreme pH, pathogenic bacteria, and bacteriophages, collectively compromising cellular viability and metabolic output; microalgae-based engineered living materials are constructed by immobilization of microalgal cells using bioactive matrices preserving cellular viability and metabolic activity; 3D bioprinting methodologies include extrusion-based printing, light-projection-based printing, and inkjet-based printing. Customized architectures of microalgae-based engineered living materials are constructed using 3D bioprinting, allowing precise spatial control over cellular distribution. Green clavate figures represent microalgae. Red oval figures represent another microorganism.

The capacity of 3D bioprinting to fabricate customized three-dimensional structures addresses the specific physiological requirements of diverse cellular systems, enabling precise regulation of scaffold morphology and microenvironmental parameters to optimize biological functions [18,19]. This technological advancement offers particularly promising methodologies for microalgae immobilization through biocompatible matrices that preserve cellular viability and metabolic activity in open environments (Figure 1). The synergistic integration of living cells with structural biomaterials, collectively termed engineered living materials (ELMs), creates novel functional composites [20]. ELMs derive functionality from cellular metabolism, while the scaffold microenvironment sustains essential cellular physiological activity through optimized gas exchange, nutrient diffusion, and light transmission [21,22].

Specifically, microalgae-immobilizing biomaterials were defined as microalgae-based engineered living materials (MB-ELMs), which enable the construction of species-specific cultivation systems through strategic incorporation of photosynthetic cells within tailored matrices. Beyond basic immobilization, spatial patterning emerges as a critical field in biomaterial science [25,26], where bioprinting’s resolution capabilities directly overcome the spatial constraints inherent in conventional microalgae applications. By combining material engineering with spatial control, several innovative applications are pioneered ranging from controlled biosynthesis platforms to advanced ecological remediation systems. The integration of 3D bioprinting with microalgal biotechnology enables native microalgal capacities, significantly expanding potential applications for MB-ELMs. This technological advancement marks a shift in microalgae research, transitioning from traditional suspension cultures to spatially organized, functionally enhanced biohybrid systems [27]. This review systematically summarizes the emerging paradigm of 3D bioprinting applications for MB-ELMs, focusing on technological innovations that bridge materials science with microalgal biotechnology to unlock new functional capabilities.

## 2. History and Progress of Microalgae-Based 3D Bioprinting

In 2015, Gelinsky’s group pioneered the development of microalgae-laden hydrogel scaffolds via 3D bioprinting. Lode et al. employed multi-nozzle 3D printing to co-encapsulate *Chlamydomonas reinhardtii* and human SaOs-2 cells within alginate/methylcellulose matrices, achieving sustained co-cultivation viability for 24 h. While observing a reduction in SaOs-2 cell count, this landmark study introduced the novel concept of green bioprinting, successfully demonstrating the feasibility of integrating oxygen-producing microalgae with mammalian cells within a unified scaffold [28]. In the same year, Krujatz et al. extended this methodology to two microalgal species, *C. reinhardtii* (11.32b) and *Chlorella sorokiniana* (UTEX 1230), through precise 3D bioprinting in alginate/methylcellulose hydrogels, revealing microalgal growth rates of 0.4–0.7 d^−1^ under varied temperatures (26 °C, 30 °C, and 37 °C) and illumination regimes (continuous versus 14/10 h light/dark cycles). Notably, immobilized microalgae exhibited superior growth performance at 37 °C under cyclic illumination compared to free-floating counterparts in suspension cultures [29]. In 2023, Windisch et al. first advanced this technology for space applications by developing 3D-bioprintable alginate/methylcellulose hydrogels containing *Chlorella vulgaris* CCALA 269. These formulations retained bioprinting capacity after 28-day cold storage at 4 °C, with cryopreserved samples maintaining photosynthetic efficiency [Y (II) = 0.31–0.33] comparable to non-cryopreserved controls [Y (II) = 0.35] [30]. This breakthrough positions prefabricated microalgal bioinks as promising oxygen-generation systems for sustainable life support in space stations. Additionally, from 2020 to 2025, research on microalgae-based 3D bioprinting gradually emerged in scientific publications. These studies will be systematically categorized and discussed in Section 4 of this review.

An analysis of the existing studies reveals that microalgae-based 3D bioprinting technology has undergone an evolutionary trajectory from fundamental biocompatibility verification to functionalized system integration. This review categorizes this evolutionary process into three distinct phases: foundational forming, functional development, and intelligent systems. During the foundational forming stage, the key technological challenge involved validating microalgal viability under shear stress conditions, though long-term stability of embedded microalgae remained problematic. The functional development phase primarily focused on formulating novel bioinks designed to enhance and confer specific properties to microalgae. Transitioning to the intelligent systems stage, research emphasis shifted toward self-responsive materials, where genetically engineered microalgae enabled environmentally adaptive structural transitions and specialized functionalities. Microalgae-based 3D bioprinting presents a viable solution addressing the spatial inefficiency intrinsic to conventional microalgae cultivation methods. Moreover, 3D-printed MB-ELMs exhibit dual capabilities of carbon sequestration and autonomous growth, positioning them as promising candidates for developing carbon-neutral material systems. While 3D bioprinting of microalgae shows considerable application potential, the overall research remains confined to laboratory-scale studies. Key challenges that need to be addressed are discussed in detail in Section 5.2.

Beyond living cell application, emerging research explores 3D printing utilization of microalgal biomass [31,32,33,34,35,36,37,38]. In these studies, microalgae were incorporated in biomass form into 3D printing inks to enhance or confer specific functionalities to the ink. This review specifically focuses on viable cell-based 3D printing systems.

## 3. Crucial Technical Components of Microalgae-Based 3D Bioprinting

### 3.1. Matrices of Microalgae-Based 3D Bioprinting

Hydrogel systems constitute the principal matrix platform for microalgae-based 3D bioprinting, with alginate, starch, and carrageenan emerging as predominant choices due to the structural adaptability and biological compatibility [39]. Hydrophilic polymer networks demonstrate exceptional water retention capacity while permitting essential nutrient diffusion and cellular communication [40]. A study by Balasubramanian et al. established the viability of alginate-based hydrogel scaffold for bioprinting *C. reinhardtii* CC-124 cells on bacterial cellulose substrate. Under normal laboratory conditions (room temperature, a 12/12 h light/dark cycle), the encapsulated microalgae cells maintained stable proliferation for over four weeks, evidenced by sustained chlorophyll content and cell density. Remarkably, nutrient deprivation for 72 h caused no measurable decline in these viability indicators [41]. The structural superiority of alginate metrices lies in the capacity to facilitate light penetration, gas exchange (O_2_/CO_2_), and nutrient transport, three critical parameters for photosynthetic microorganisms [42,43,44]. Ionic crosslinking enables precise spatial control during scaffold fabrication, cementing alginate’s status as a crucial material in 3D bioprinting applications.

Despite these advantages, hydrogel-based MB-ELMs face two critical limitations: compromised mechanical integrity and finite functional durability. The inherent viscoelastic properties of hydrogels restrict the load-bearing applications, while reversible ionic crosslinking in physiological environments permits gradual microalgal leakage from alginate matrices [45]. Photopolymerization-mediated crosslinking creates permanent covalent networks through radical-induced polymerization of light-sensitive polymers [46]. When exposed to specific wavelengths, photoinitiators generate reactive species that catalyze interchain bonding, transforming the hydrogel’s viscoelastic profile. This process elevates the storage modulus (*G*’), a key indicator of elastic solid behavior, from transient gel states to robust polymer networks. Dranseike et al. demonstrated this principle by incorporating lithium phenyl-2,4,6-trimethylbenzoylphosphinate (LAP) into a Pluronic F127 hydrogel, achieving unprecedented MB-ELM longevity exceeding 400 days through UV-induced crosslinking [47]. However, cytotoxic effects of synthetic photoinitiators necessitate cautious implementation [48]. An innovative approach by Vazquez-Martel et al. circumvented this limitation by functionalizing microalgal-derived triglycerides and chlorophyll as endogenous photoactive components, enabling scaffold fabrication without an exogenous initiator [49]. This biomimetic strategy opens new avenues for sustainable material development in algal bioprinting.

Alternative material systems are being investigated to address hydrogel limitations. Pozzobon et al. conducted cytotoxic screening of common 3D printing polymers (Acrylonitrile Butadiene Styrene, ABS; Polycarbonate Blend, PC-Blend; Polylactic Acid, PLA; Acrylate Resin) on *Chlorella vulgaris*, identifying PLA as the sole material maintaining baseline reactive oxygen species (ROS) levels [50]. Hybrid hydrogel composites show particular promise. Zhao et al. developed a silk fibroin/hydroxypropyl methylcellulose matrix support for *Platymonas* proliferation for 28 days with 90-day photosynthetic activity retention [51]. An advanced functionalization strategy was demonstrated by He et al., who presented a supramolecular hydrogel combining thixotrophic hyaluronic acid with a cucurbit [8] uril (CB [8]) macrocycle. UV post-processing of this system induced ten-fold *G*’ enhancement (8000 Pa), while the resultant *Bacillus*–*Chlorella* co-culture system demonstrated efficient bioremediation of acrylamide and methyl orange [52]. These developments highlight the critical interplay between material innovation and biological functionality in MB-ELM engineering.

### 3.2. Methods of Microalgae-Based 3D Bioprinting

Three-dimensional bioprinting enables precise architectural customization of scaffolds to accommodate species-specific microalgal requirements and functional objectives. This technology further permits functional modification of bioinks during MB-ELM fabrication to optimize microenvironmental conditions for cellular proliferation [53]. Current bioprinting paradigms primarily employ three technical approaches: extrusion-based [54], light-projection-based [55], and inkjet-based systems [56]. Recent studies on microalgae-based bioprinting predominantly focus on extrusion-based methods, while emerging light-projection technologies show particular promise for mimicking natural microenvironments at cellular scales. Inkjet systems offer specific applications in bioelectronic integration. The choice of methodology ultimately depends on the specific balance between resolution, biocompatibility, and functional requirements for target MB-ELM applications. Table 1 provides a clearer comparative visualization of the three bioprinting methods.

#### 3.2.1. Extrusion-Based Bioprinting

**Figure 2:**
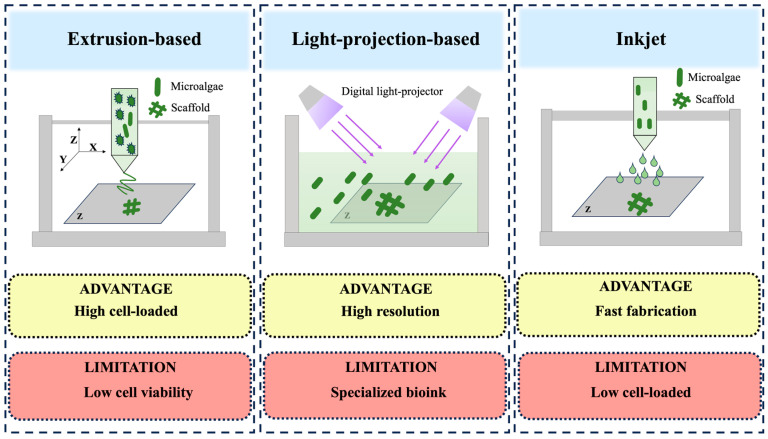
Schematic representations of 3D bioprinting methods. Extrusion-based printing: the bioink is loaded into the bioprinter prior to printing. The printer moves along three-dimensional axes (X, Y, and Z), depositing the bioink onto the printing platform through mechanical extrusion according to a predefined model, resulting in layer-by-layer fabrication. During this process, the extrusion of bioink is continuous. It is noteworthy that cells in the printer experience diminished viability due to compressive mechanical stress. Light-projection-based printing: the printing platform is immersed in a reservoir of bioink, and a light projector selectively solidifies the bioink layer by layer following a predetermined pattern to achieve the desired structure. Inkjet printing: the bioink preloaded into the bioprinter is ejected onto specified locations in accordance with a pre-designed three-dimensional model to facilitate precise construct formation.

Extrusion-based bioprinting, the most widely adopted method for microalgae-based systems, employs viscous bioinks containing living cells. Pre-prepared bioink is stored in the printer and mechanically extruded through a nozzle onto a printing platform (Figure 2). Precise control of printer parameters (e.g., temperature, pressure, and deposition speed) minimizes adverse effects on cell viability while maintaining bioink fluidity during printing [63]. The method enables the processing of bioinks with high cell densities (>1 × 10^7^ cells/mL). However, the inherent shear stress generated during mechanical extrusion compromises cellular viability. The post-extrusion solidification mechanism of bioinks inherently limits the resolution (100–500 μm), rendering it suitable primarily for constructs where high precision is nonessential. Post-printing solidification of printed structures is commonly achieved through ionic crosslinking and photo-crosslinking. The extrusion-based fabrication method endows the printed structure with high mechanical strength. Final print quality depends critically on bioink properties such as viscosity, rheological behavior, and shear-thinning characteristics [64]. Although pressure-assisted systems offer full parameters control, prolonged printing durations may reduce cell viability due to cumulative mechanical and thermal stress.

The accessibility of extrusion-based bioprinting, requiring relatively simple equipment and biomaterials, has driven its predominance in current research. Balasubramanian et al. demonstrated the feasibility of this approach by developing recyclable *C. reinhardtii*-laden alginate hydrogel, where immobilized cells maintained stable growth for a long time [41]. Oh et al. further advanced this methodology by fabricating an alginate hydrogel scaffold with complex geometries, optimizing the surface area-to-volume ratio to enhance photosynthetic efficiency [65]. These studies highlight the strategic focus on maximizing scaffold surface area to improve light penetration and gas exchange, a common design principle in microalgae bioprinting.

#### 3.2.2. Light-Projection-Based Bioprinting

Light-projection-based bioprinting utilizes photosensitive materials exposed to UV light patterns for layer-by-layer polymerization, enabling extremely high resolution (<50 μm). During printing, the platform is submerged in a photopolymerization resin, and a digital light projector selectively cures the material according to predefined 3D models (Figure 2). This technique achieves rapid fabrication speeds while minimizing cell exposure to non-physiological conditions. Its high resolution and structural precision make it ideal for creating intricate biomimetic architecture [66,67].

Despite these advantages, the technology faces material-specific challenges. Photopolymerizable resins must maintain biocompatibility while supporting microalgal growth, and prolonged UV exposure during multilayer printing may compromise cell viability. Wangpraseurt et al. replicated coral tissue microstructures using light-projection printing, achieving microalgae densities of 10^9^ cells/mL through optimized light management strategies [66].

#### 3.2.3. Inkjet-Based Bioprinting

Inkjet-based printing operates on a non-contact principle, depositing bioink droplets onto predetermined locations through piezoelectric or thermal actuators (Figure 2). This method allows customizable parameter adjustments for diverse environmental conditions and achieves high resolution (50–300 μm) [68]. Inkjet-based bioprinted structures typically undergo solidification via thermal treatment (thermosensitive gelation) and ionic crosslinking. Using inkjet-based low-viscosity bioinks limits mechanical strength compared to extrusion-based methods.

However, inkjet-based printing struggles with low cell-loading capacity. Furthermore, post-printing steps such as chemical or photopolymerization pose challenges for maintaining cell viability. Sawa et al. demonstrated the functional potential of this approach by printing *Synechocystis* sp. PCC 6803 cells onto carbon nanomaterials, creating a biophotovoltaic thin-film cell capable of generating stable current for over 100 h [69].

## 4. Application of Microalgae-Based 3D Bioprinting

The strategic integration of microalgae within three dimensional scaffolds via 3D bioprinting creates favorable conditions for optimal light penetration and nutrient distribution, effectively sustaining the viability and metabolic functions of immobilized microalgae over extended periods. This advanced manufacturing technique substantially broadens the application scope of microalgae-loaded scaffolds across multiple disciplines [53,70]. The synergistic combination of photosynthetic microorganisms with additive manufacturing technology demonstrates broad interdisciplinary application potential: it not only improves bioenergy conversion efficiency through enhanced carbon sequestration and photoelectrochemical performance but also unlocks novel implementation pathways in emerging fields such as living building materials, biophotovoltaic systems, photosynthetic biosynthesis, bioremediation, tissue engineering, and food engineering. The subsequent sections will provide a systematic discussion on the applications of 3D bioprinting technology with microalgae in these fields.

### 4.1. Microalgae-Based 3D Bioprinting in Living Building Materials

#### 4.1.1. Living Building Materials

**Figure 3:**
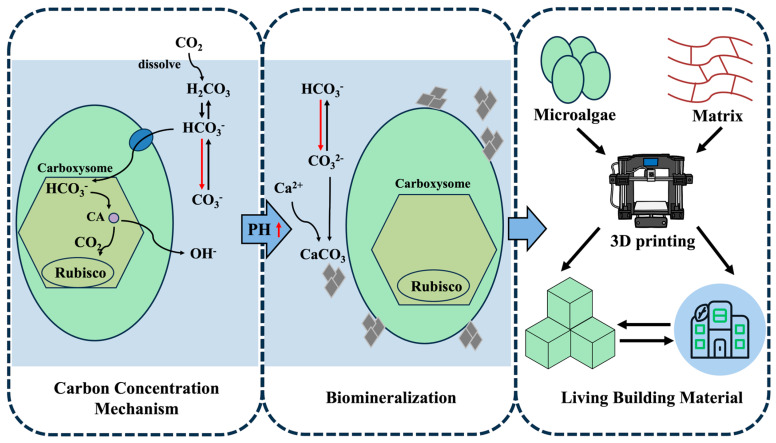
Schematic representations of 3D-bioprinting-enabled LBMs. Microalgae are capable of CO_2_ sequestration through the carbon concentration mechanism during photosynthesis using dissolved CO_2_ as the major carbon source creating localized pH elevation of the microenvironment. The combination of calcium ions with carbonate ions is induced by an alkaline shift in the microalgal cell microenvironment, resulting in calcium carbonate precipitation on and around microalgal cell surfaces. Immobilized within bioactive matrices, microalgae can enhance material mechanical strength via biomineralization. Coupled with 3D printing’s capacity for customized structural fabrication, microalgae-based engineered living materials can be used to construct living building materials.

The escalating global carbon crisis has intensified demand for innovative carbon mitigation strategies, with microalgae-based systems emerging as potent solutions due to the superior photosynthetic efficiency and carbon sequestration capacities [71]. The construction industry contributes 39% of global anthropogenic carbon emissions, including 11% from concrete production alone [72]. Living building materials (LBMs) utilize microorganisms to produce construction materials that exhibit mechanical and biological properties [73]. Microalgae-based LBMs are supposed to diminish carbon emissions of construction industry through microalgal biomineralization within matrices. Microalgae utilize carbon concentration mechanisms (CCMs) alongside biologically regulated mineralization processes to achieve efficient atmospheric carbon conversion. Additionally, calcium carbonate (CaCO_3_) is produced by microalgae through biomineralization, which refers to organism-mediated deposition of inorganic minerals through controlled interactions between biomacromolecules, metabolic pathways, and environmental parameters [74]. The microalgae immobilized within the matrix enhance the mechanical strength of structure by fixing atmospheric carbon dioxide in the form of CaCO_3_ through their physiological processes, demonstrating great potential for developing self-repair material (Figure 3).

#### 4.1.2. Three-Dimensional-Bioprinting-Enabled LBMs

Current research focuses on optimizing the mechanical performance of microalgae-based LBMs, which currently exhibit lower tensile strength compared to conventional concrete. A promising strategy involves augmenting CaCO_3_ deposition through enzymatic enhancement. Microalgae naturally secrete carbonic anhydrase (CA), an enzyme that catalyzes CO_2_ hydration to accelerate mineral precipitation within hydrogel matrices, effectively reinforcing material integrity [75]. Heveran et al. presented a dual-functional LBM incorporating *Synechococcus* sp. PCC 7002, where a temperature/humidity-responsive hydrogel scaffold achieved three critical functions: maintaining cyanobacterial viability (9–14% survival after 30 days at >50% humidity), enabling structural self-repair, and enhancing compressive strength via regulated biomineralization [76]. Parallel work by Zhu et al. with *Synechococcus* PCC 8806 established foundational dynamics and load-bearing performance under simulated service conditions [77]. It is noteworthy that *synechococcus* sp. PCC 7002 is a common strain of marine microalgae. All microalgae mentioned in this section are marine species.

The advent of 3D bioprinting addresses critical challenges in urban construction by enabling rapid prototyping of structurally optimized, algae-integrated architectures. Reinhardt et al. engineered a bio-concrete through extrusion-based bioprinting of *Synechococcus* sp. PCC 7002 within alginate/methylcellulose hydrogels. Strategic incorporation of sand particles (30–50 wt%) served dual purposes as nucleation sites for biomineralization and mechanical stabilizers, yielding compressive strengths up to 50.1 ± 6.3 MPa in sand-enriched formulations, a 6.6-fold enhancement compared to the algae-free counterpart. Crucially, the printed grid architecture enhanced gas/nutrient exchange while maintaining microalgal viability, demonstrating synergistic integration of biological and structural functions [78]. Dalia et al. employed projection-based bioprinting to create *Synechococcus* sp. PCC 7002 embedded lattices (0.15 to 0.70 mm pore geometry) optimized for concurrent carbon capture and structural performance. The dual-phase carbon sequestration profile, initial carbonate precipitation (2.2 ± 0.9 mg CO_2_/g substrate as insoluble carbonates over 30 days) followed by sustained mineralization (26 ± 7 mg CO_2_/g substrate across 400 days), preliminarily demonstrates scalable carbon-negative construction potential through continuous atmospheric carbon conversion [47].

#### 4.1.3. Future Perspectives of 3D-Bioprinting-Enabled LBMs

Microalgae-based LBMs are envisioned not merely as structural components but as functional elements that transform buildings from carbon-emission elements into environmental modulators. Integrating microalgae LBM into non-load-bearing architectural sections could offset partial carbon emissions. While current research prioritizes the elucidation of biomineralization mechanisms, bioprinting optimization, and process parameter refinement, several critical barriers impede practical implementation, including the following: (1) achieving spatiotemporal control over mineralization uniformity and kinetics to ensure structural consistency; (2) synchronizing biological processes with printing parameters for dimensional accuracy; (3) establishing standard protocols for biosafety and lifecycle cost assessments; and (4) overcoming species-specific variability in mineralization efficiency through targeted strain engineering. Addressing these limitations requires concerted efforts in three domains: fundamental studies of biomineralization regulatory networks, technological integration of additive manufacturing with biological systems, and the development of scalable production workflows for industrial adoption.

### 4.2. Microalgae-Based 3D Bioprinting in Biophotovoltaic System

#### 4.2.1. Biophotovoltaic System

The development of sustainable energy infrastructure represents one of humanity’s most pressing technological challenges. With solar irradiance reaching the Earth’s surface annually surpassing global energy demands by four orders of magnitude, solar energy conversion has emerged as a critical scientific field [79]. Biophotovoltaic (BPV) systems leverage photogenerated electrons from photosynthetic organisms during light-dependent reactions, directing these charge carriers through engineered bioelectrochemical pathways to generate electrical current. This innovative paradigm offers a carbon-neutral alternative to conventional power generation. Microalgae have become pivotal in BPV research due to the remarkable photosynthetic quantum efficiency, rapid scalability, and ecological sustainability [80]. Distinct from traditional microbial fuel cells, microalgae-based photobioelectrochemical configurations demonstrate promising energy conversion methodologies through oxygenic electron donation mechanisms [81]. Conventional BPV fabrication via gravity-mediated cell sedimentation onto electrodes suffers from inherent scalability limitations including excessive liquid phase requirements, protracted processing durations, and restricted spatial resolution in electrode architecture. Modern bioprinting technologies circumvent these constraints through rapid prototyping of customized structure with micrometer-scale precision, demonstrating revolutionary potential for advanced BPV engineering [80,82,83]. Current research indicates that 3D bioprinting technology primarily advances BPV systems through two strategies: the fabrication of electrodes and the construction of microalgae composites.

#### 4.2.2. Strategies of 3D-Bioprinting-Enabled Biophotovoltaic Systems

##### Fabrication of 3D-Printed Electrodes

**Figure 4:**
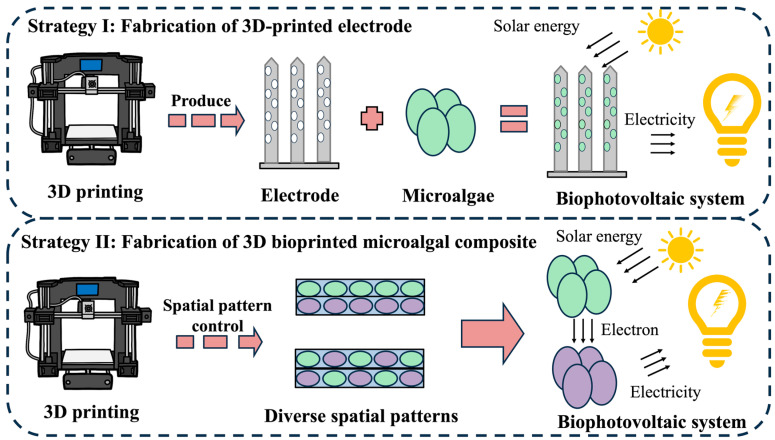
Strategies of 3D-bioprinting-enabled biophotovoltaic systems. Strategy I: the integration of microalgae cells with 3D-printed porous transparent electrodes enhances electron capture efficiency in biophotovoltaic systems, improving biophotovoltaic systems’ power output. Strategy II: diverse spatial patterns of microalgae and electrogenic bacteria encapsulated within bioactive matrices can be controlled by 3D printing. Precise spatial pattern control of microbial consortia improves inter-species electron transfer efficiency, boosting the power output of biophotovoltaic systems. Purple circle figures represent electrogenic bacteria (e.g., *Shewanella oneidensis*).

Current limitations in electron transfer efficiency within conventional BPV devices primarily stem from suboptimal photoelectron harvesting at biotic–abiotic interfaces. Hierarchical nano-porous electrode designs address this challenge through enhanced interfacial charge transfer (Figure 4). Wenzel et al. documented two-order-of-magnitude current enhancement in *Synechocystis* sp. PCC 6803 photovoltaic systems utilizing indium tin oxide (ITO) nanostructured electrodes. Chen et al. pioneered 3D-printed ITO micro-pillar arrays featuring multiscale porosity. These engineered electrodes achieved unprecedented photocurrent density (245 μA/cm^2^), approaching theoretical maximum values under standard illuminations [84]. The synergistic combination of hierarchical porosity and high surface-area-to-volume ratios in 3D-printed nanoelectrodes dramatically expands biotic–abiotic contact interfaces, thereby accelerating photoelectron transfer kinetics and enabling efficient semi-artificial photosynthetic processes. Joshi et al. developed a biomimetic mushroom-like architecture via 3D-printed graphene nanoribbon (GNR)–*Anabaena* hybrids, where anisotropic cellular alignment enhanced photocurrent output eightfold relative to isotopic controls. The dense GNR network facilitated efficient photoelectron extraction from algal photosynthetic machinery, highlighting the critical importance of spatial organization in bioenergy system optimization [85].

##### Fabrication of Bioprinted Microalgal Composite

Sawa et al. engineered an inkjet-printed bioanode through direct deposition of PCC 6803 onto carbon nanomaterials, subsequently laminating hydrogel electrolytes to construct integrated BPV devices. The dual-function hydrogel matrix served as both an ionic conductor and nutrient delivery system, sustaining continuous current generation for more than 100 operational hours [69]. Bioprinting enables sophisticated engineering of symbiotic biohybrids through spatially controlled biological integration. Liu et al. constructed alginate-encapsulated PCC 6803/*Shewanella oneidensis* MR1 consortia via 3D bioprinting, maintaining physical segregation while enabling metabolic cross-feeding. This system achieved peak outputs of 62 µW and 287 µA through reciprocal substrate exchange: MR1 metabolized algal exudates while providing photosynthetic substrates (CO_2_/H_2_O). Although power attenuation occurred over multi-day operation, this approach effectively addresses sustainability limitations in conventional BPV configurations [86].

#### 4.2.3. Future Perspectives of 3D-Bioprinting-Enabled BPV System

Bioprinting implementation in microalgal BPV systems enables spatial organization and microenvironmental customization. Precision deposition of microalgae into columnar or stratified architectures maximizes photon utilization through uniform irradiance distribution, effectively mitigating photosynthetic inefficiencies caused by cellular self-shading. Simultaneous integration of cells into conductive matrices minimizes electron transfer distances at biotic–abiotic junctions, significantly reducing energy dissipation. A critical distinction from other bioprinting applications lies in essential requirement for multifunctional bioinks combining cytocompatibility, mechanical durability, and electrical conductivity—a substantial challenge given the inherently insulative nature of hydrogel matrices. Future matrix innovations will likely focus on nanocomposite formulations incorporating carbon nanomaterials or metallic dopants to achieve optimal bioelectronic interfaces performance.

### 4.3. Microalgae-Based 3D Bioprinting in Photosynthetic Biosynthesis

#### 4.3.1. Photosynthetic Biosynthesis

**Figure 5:**
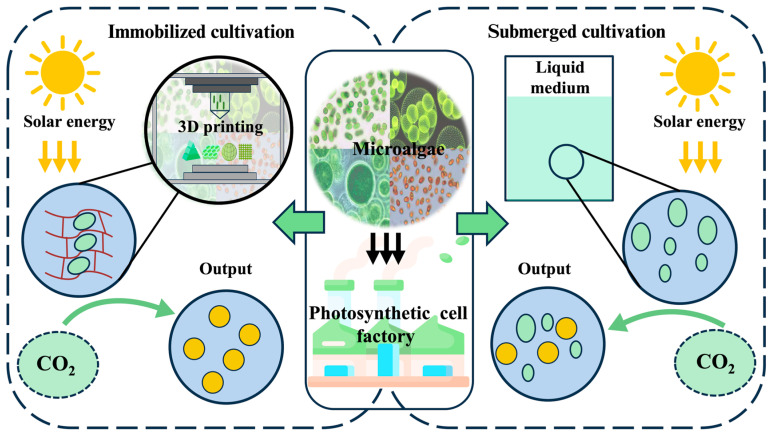
Schematic representations of 3D-bioprinting-enabled photosynthetic biosynthesis. Microalgae immobilized within bioactive matrices harness light energy to sequestrate CO_2_ through photosynthesis. Rewiring of microalgae’s metabolic networks redirects carbon flux toward targeted pathways, enabling sustainable production of high-value outputs. Additionally, immobilization of microalgal cells within bioactive matrices significantly streamlines downstream processing, achieving higher product recovery yields while maintaining cellular viability and metabolic activity for extended production cycles. Furthermore, 3D-printed customizable scaffolds further enhance process adaptability across diverse production applications. Yellow circle figures represent products of microalgae metabolism. Green circle figures represent microalgae cells.

Microorganism-mediated biosynthesis has established itself as a cornerstone technology for producing bioactive macromolecules. Within this domain, microalgae have emerged as particularly promising cellular platforms for synthesizing high-value compounds ranging from renewable fuels to specialty carbohydrates. The engineering of advanced microalgal cell factories currently represents a pivotal research trajectory in microalgal biotechnology [87,88]. Conventional biosynthesis methodologies typically rely on microbial proliferation in nutrient-rich media, followed by subsequent separation and purification of target metabolites from the cellular–medium matrix. However, conventional liquid-phase microalgal cultivation faces fundamental technological constraints. The inherent self-shading effect in submerged systems attenuates light transmission via combined photon absorption and scattering effects in aqueous suspensions, especially at elevated biomass concentrations [89]. This photonic restriction adversely impacts both photosynthetic quantum yield and metabolic flux. Furthermore, suspended cultures exhibit increased vulnerability to microbial contamination while requiring extracellular separation processes for product harvest. These constraints have catalyzed several innovations in immobilized microalgal cultivation systems, where cells are embedded within polymer matrices (Figure 5). This paradigm not only modulates cellular proliferation dynamics but also amplifies biocatalytic performance. The encapsulated microorganisms maintain metabolic activity over extended durations, enabling continuous production cycles while dramatically simplifying post-cultivation purification processes [90,91].

#### 4.3.2. Three-Dimensional-Bioprinting-Enabled Photosynthetic Biosynthesis

The immobilized cultivation framework represents a promising methodology for industrial implementation [92]. Bioprinting integration has transformed the conventional immobilization approach through a vertically structured cultivation architecture, effectively resolving spatial and resource inefficiency characteristics of traditional liquid systems. Modern bioprinted microalgal cell factories utilize precisely engineered 3D matrices that optimize both microbial viability and metabolic productivity [91]. This advanced manufacturing strategy ensures scalable output while enabling streamlined product harvesting. Current innovations include customizable scaffold configurations tailored to specific cellular requirements through adjustable geometries and bioink formulation. A notable advancement by Szilveszter et al. features a novel hydrogel material composed of alginate/galactoglucomannan methacrylate (GGMMA) and lithium phenyl-2,4,6-trimethylbenzoylphosphinate (LAP), which enables microalgae encapsulation via 3D printing without ionic crosslinking. Trimethoxysilylpropyl methacrylate (TMPSM) functionalized glass substrates supporting thin-film bioreactors demonstrated multifunctional capabilities: *Synechocystis* sp. PCC 6803 achieved ethylene biosynthesis (780 μmol mgChl^−1^ under 35 μmol photons m^−2^ s^−1^), while *C. reinhardtii* mediated cyclohexanone conversion to ε-caprolactone with 87% efficiency. Mechanistically, bioprinted constructs exhibited enhanced robustness (*G*’ = 10–20 kPa, shear stress = 390–1000 Pa) compared to conventional ionic gels (*G*’ = 1–2 kPa, shear stress = 40–75 Pa). Remarkably, matrix-immobilized cultures matched free-floating systems in specific activity (3.46 ± 0.2 vs. 3.6 ± 0.8 U gDCW^−1^), surpassing traditional methods with two- to four-times higher ethylene production [46].

#### 4.3.3. Future Perspectives of 3D-Bioprinting-Enabled Photosynthetic Biosynthesis

Three-dimensional-bioprinting-enabled photosynthetic biosynthesis demonstrates great potential in sustainable cell factory development through spatially optimized and mass-transfer-enhanced cellular scaffolds, achieving precise control over microbial spatial organization and microenvironmental parameters, significantly augmenting production efficiency [27]. Precision-printed algal scaffolds optimize cellular light distribution patterns, effectively mitigating the heterogenous photon delivery inherent to liquid cultures. The geometrically enhanced light-harvesting capacity accelerates photosynthetic kinetics, thereby boosting biosynthesis rates. Future research may focus on employing 3D bioprinting techniques to engineer microalgae-containing synthetic microbial consortia for photosynthetic cell factories. The bioprinted scaffolds, featuring spatially organized microalgae and heterotrophic species within biomaterial matrices, could establish partitioned metabolic pathways for enhanced biosynthesis. Nevertheless, technical challenges persist regarding cellular stress mitigation, production yield optimization, manufacturing cost reduction, and long-term operation stability. Future advancements demand synergistic integration of microbial system biology, precision metabolic engineering, and smart biomaterial development, complemented by holistic optimization of scalability and economic viability.

### 4.4. Microalgae-Based 3D Bioprinting in Bioremediation

#### 4.4.1. Bioremediation

**Figure 6:**
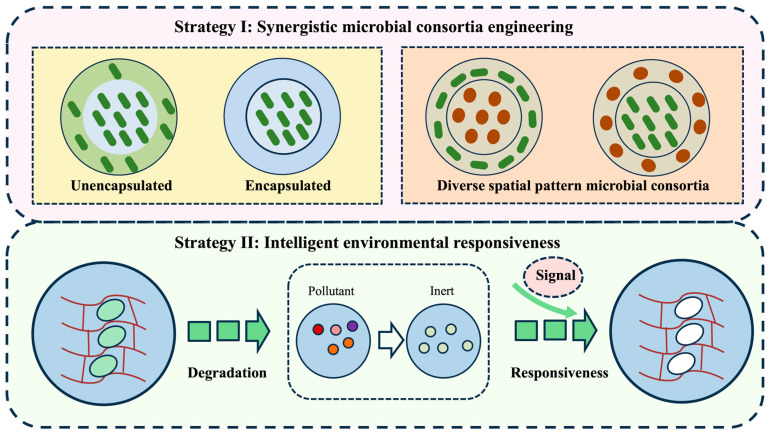
Strategies of 3D-bioprinting-enabled bioremediation. Strategy I: immobilization of microalgae within matrices prevents leakage of microalgae. The bioink of heterogeneous composition enables co-immobilization of microalgae with functional bacteria to construct spatially organized microbial consortia, where the collective functionality of the microbial community facilitates bioremediation. Strategy II: genetic engineering of microalgal cells allows the immobilized cells to respond to environmental signaling molecules. This engineered capability can either trigger specific metabolic pathways to secrete target metabolites or induce programmed cell death when required. Red circle figures represent functional bacteria (e.g., *Bacillus subtilis*).

Anthropogenic emissions of heavy metals, organic pollutants, and greenhouse gases from rapid industrialization have created critical environmental burdens requiring urgent remediation solutions [93]. Bioremediation technologies leveraging microalgal biosorbents offer ecologically compatible alternatives for pollutant mitigation. The cellular architectures of microalgae feature diverse functional moieties including carboxyl, sulfate, hydroxyl, and phosphate groups that confer specific binding affinities for contaminant adsorption [94]. Immobilized microalgal matrices present a promising solution by creating protective microenvironments that sustain algal viability while preventing ecosystem contamination [95]. Immobilization of microalgae within a matrix not only harnesses the pollutant-degrading capabilities but also prevents leakage of microalgae, thereby avoiding potential microbial biohazards (Figure 6). Bioprinting technology provides a feasible paradigm through precision-engineered bio-scaffolds with tailored structural and functional characteristics, synergizing ecological restoration principles with advanced manufacturing precision. Current studies demonstrate that 3D bioprinting technology primarily enhances bioremediation efficacy through two practical strategies: constructing synergistic microbial consortia and developing intelligent-responsive printed architectures.

#### 4.4.2. Strategies of 3D-Bioprinting-Enabled Bioremediation

##### Construction of Synergistic Microbial Consortia

Jiang et al. developed retrievable microalgae hydrogel networks (MHNs) by confining living *Chlorella zofingiensise* in double-network hydrogel. Coating MHNs with tannic acid (MHN@TA) creates a semipermeable membrane that prevents leakage of microalgae. MHN@TA largely maintained promising degradation efficiency even at 400 mg/L of tetracycline [96]. While individual microalgal species exhibit constrained remediation capabilities, engineered consortia demonstrate enhanced degradation potential for complex contaminants. He et al. developed a dual-network hydrogel supporting 3D bioprinted *Chlorella vulgaris-Bacillus subtilis* communities, establishing symbiotic pollutant degradation pathways. Bacterial mineralization of organic substrates generated CO_2_ for algal photosynthesis, while reciprocal oxygen exchange sustained bacterial respiration. Degradation kinetics revealed approximately 70% methyl orange removal within 12 h and 90% compound elimination within 48 h, demonstrating efficient contaminant cycling [52]. Ou et al. from the same group advanced this concept through microfluidic fabrication of gelatin/methacrylated gelatin-carboxymethyl cellulose scaffolds with compartmentalized microbial niches. Spatial segregation of *C. vulgaris* and *B. subtilis* populations achieved 90% amoxicillin removal (300 mg/L) with concurrent methyl orange degradation (15%, 100 mg/L) within 24 h, while minimizing microbial competitions [97]. Sun et al. further optimized this strategy through coaxial 3D-printed alginate/Pluronic 127 hydrogel fibers, encapsulating *C. vulgaris* shell phase and *B. subtilis* in the core region for simultaneous methyl orange/molybdenum remediation [98].

##### Intelligent Environmental Responsiveness

Advanced bioprinting enables the creation of stimulus-responsive bioremediation systems through synthetic biology integration. Datta et al. engineered riboswitch-mediated genetic circuits in *Synechococcus elongatus* PCC 7942 embedded within alginate hydrogels, creating environmentally adaptive scaffolds. The Laccase + riboF-Lysis + strain demonstrated 72.8% ± 6.8% indigo carmine dye decolorization over 10 days versus 3.8% ± 1.0% in controls, coupled with programmable cell lysis via theophylline induction to prevent ecological dispersal [99]. This dual functionality of contaminant degradation and controlled biocontainment represents a paradigm shift in sustainable bioremediation technologies.

#### 4.4.3. Future Perspectives of 3D-Bioprinting-Enabled Bioremediation

Real-world contamination scenarios present complex multipollutant interactions that current single-contaminant studies inadequately address. Industrial effluents and contaminated soils frequently contain synergistic pollutant mixtures that complicate remediation kinetics and strain selection. Future research priorities could encompass the following: (1) mechanistic understanding of cross-contaminant interactions; (2) optimization of algal–bacterial consortia for complex matrices; (3) spatiotemporal orchestration of microbial consortia through 3D bioprinting. The technology’s capacity for controlling spatial patterning offers particular microbial community architectures, enabling targeted pollutant degradation cascades. Current limitations in handling multicomponent contamination systems necessitate development of intelligent scaffolds with real-time environmental sensing and adaptive metabolic responses. Bridging laboratory-scale innovations to field applications will require multidisciplinary integration of synthetic biology, materials science, and environmental engineering to achieve robust, scalable bioremediation solutions.

### 4.5. Microalgae-Based 3D Bioprinting in Tissue Engineering

#### 4.5.1. Tissue Engineering

The convergence of bioprinting technology with tissue engineering has catalyzed a novel research paradigm in microorganism-driven biomaterial innovation [53]. Oxygen from photosynthesis is critical for cellular metabolism and energy generation, particularly in thick tissues (>300 μm) or in the absence of vascularization, where conventional artificial tissues frequently succumb to hypoxic necrosis and functional impairment [100]. Microalgae-derived photosynthetic oxygen generation offers a sustainable biological strategy to address this critical limitation. Emerging studies have validated microalgal biomaterial application regenerative contexts [101,102,103,104], while bioprinting integration amplifies these benefits through precision-engineered 3D microarchitectures. Spatially controlled microalgal deposition within customized scaffolds enables optimized oxygen gradient establishment, creating symbiotic microenvironments for algal–mammalian cell co-cultivation.

#### 4.5.2. Three-Dimensional-Bioprinting-Enabled Tissue Engineering

Lode et al. pioneered extrusion-based bioprinting of *C. reinhardtii*/SaOs-2 osteosarcoma cell co-cultures using alternative hydrogel layers in predefined grid configurations. While demonstrating cellular coexistence, this foundational work lacks comprehensive validation of microalgal contributions to tissue engineering outcomes. The non-physiological grid architecture and underemphasized bioprinting advantages limit translational relevance [28]. Dani et al. subsequently evaluated four microalgal species in 3D-printed matrices under mammalian culture conditions (37 °C, red light), proposing spatial orchestration of algal–mammalian cell distributions for diffusion-independent oxygen supply [105]. Maharjan et al. advanced this concept through sacrificial bioprinting of *C. reinhardtii*-laden honeycomb templates with HepG2-encapsulating hydrogels. Photosynthesis-maintained oxygenation enhanced hepatocyte viability during critical culture phases, with subsequent cellulase digestion generating perfusable microchannels. Comparative analysis demonstrated superior early-stage hepatocyte survival versus conventional cultures, validating microalgae as spatiotemporal oxygen generators [106].

Clinical translation potential emerges in hypoxic tissue regeneration models. Wang et al. developed microfluidic-assisted in situ bioprinting of *Chlorella pyrenoidosa*-encapsulated hollow fiber matrices, demonstrating accelerated wound healing via hypoxia alleviation, neovascularization, and ECM remodeling [107]. Inspired by the symbiotic structure of lichens, Liu et al. engineered a bioprinted *Chlorella zofingiensis*/*Bacillus subtilis* (*B. subtilis*) alginate/carrageenan hydrogel (BBH) for diabetic wound management. The probiotic–antimicrobial/photosynthetic–oxygenation dual system promoted fibroblast migration, HUVEC angiogenesis, and rapid re-epithelialization (90% restoration in 12 days). Transcriptomic analysis revealed upregulated keratinocyte differentiation pathways, establishing BBH’s multifunctional therapeutic efficacy [108].

#### 4.5.3. Future Perspectives of 3D-Bioprinting-Enabled Tissue Engineering

While 3D bioprinting microalgal systems show promise in addressing superficial hypoxia, deeper tissue construction demands sophisticated oxygen gradient engineering [109,110,111]. Current limitations include inadequate biomimicry of multicellular tissue complexity and uncharacterized immunological responses to algal metabolites. Future research will likely prioritize (1) the development of biosafety-optimized microalgal strains through rigorous host-integration assessments; (2) synergistic design of algae–stem cell-fibroblast tri-cultures with optimized spatial ratios; and (3) advanced biomimetic architectures replicating native tissue microenvironmental cues. Current technological capabilities remain inadequate for constructing multiscale vascularized tissues with physiologically relevant oxygen dynamics. Bridging this gap requires interdisciplinary convergence of synthetic biology, computational modeling, and multi-material bioprinting to achieve spatiotemporal control over photosynthetic oxygenation in complex tissue analogues.

### 4.6. Microalgae-Based 3D Bioprinting in Food Engineering

#### 4.6.1. Three-Dimensional Printing-Enabled Food Engineering

Microalgae, renowned for the diverse components—including functional proteins, polyunsaturated fatty acids, and essential minerals—serve as nutrient-dense functional ingredients in the development of health-promoting foods [112,113,114]. Concurrent with global shifts toward sustainable food systems, the food industry is transitioning to mass customization strategies to address personalized demands for sensory attributes (texture, flavor, color) and nutritional profiles [115]. Within this paradigm, 3D-printed foods emerge as a transformative solution capable of tailoring edible products to individual genetic predispositions, health status, and environmental considerations. Both processed microalgae biomass and live microalgae cells hold potential for integration into 3D-printed food. While microalgae biomass is commonly utilized as a functional additive to modify specific food properties, the incorporation of live microalgae cells presents greater technical complexity while offering innovative avenues for conferring dynamics biochemical functionalities.

#### 4.6.2. Three-Dimensional-Bioprinting-Enabled Food Engineering

##### Living Microalgae System

Liu et al. developed a biogenic microalgae-laden hydrogel (BMH) system via 3D-printing of alginate/carrageenan matrices encapsulating live microalgae. This architecture optimizes spatial light and CO_2_ utilization while eliminating liquid culture requirements, relying solely on a foundational agar layer to sustain growth. The BMH demonstrated enhanced productivity of proteins, carbohydrates, lipids, chlorophyll, and carotenoids compared to traditional batch cultures, achieving an 8.8-fold increase in biomass yield per water input. Remarkably, the liquid-free design enabled reversible dehydration–rehydration cycles without compromising cellular viability. Composed entirely of natural materials, the BMH system meets direct food safety standards, eliminating the need for post-processing [116]. This innovation establishes a framework for scalable, resource-efficient microbial cultivation systems in precision food manufacturing.

##### Microalgal Biomass

Post-processed microalgal biomass expands opportunities for functional food design. Microalgal biomass is used as an additive for 3D-printed food for improving functionalities of food. Uribe-Wandurraga et al. improved dough printability and thermal stability by incorporating freeze-dried microalgae, demonstrating its utility as a precursor for 3D-printed cookies [36,37]. Vieira et al. advanced this approach by encapsulating microalgae extracts within protective matrices prior to dough integration, enhancing stability during baking and storage [38]. Wang et al. demonstrated that incorporating this residual biomass into dough significantly improved the printability of cookies during 3D printing [35], while microalgal astaxanthin was used to dye the marine-macroalgae-based 3D-printed fish analogs [31].

#### 4.6.3. Future Perspectives of 3D-Bioprinting-Enabled Food Engineering

Despite this potential, critical challenges hinder commercial translation. Inherent marine-like and bitter flavor profiles reduce consumer acceptance. Textural constraints arise from the fibrous nature of algal materials, complicating meat analogue simulation and complex texture fabrication. Technical hurdles include optimizing bioink rheology for printability, ensuring post-processing edibility, and managing equipment costs, particularly for food-grade devices requiring stringent hygiene compliance.

## 5. Prospects

### 5.1. The Future of 3D Bioprinting Technology

Extrusion-based bioprinting remains the most widely adopted approach due to its equipment simplicity, broad material compatibility, and capability for constructing complex structures. However, its technological progression is constrained by inherent limitations in resolution and time-intensive fabrication processes. Ligh-projection-based bioprinting has emerged as a high-precision alternative, enabling the fabrication of intricate microstructures through photopolymerization. Despite these advantages, technical complexities in multi-material integration and photopolymerization-induced cellular stress restrict its practical implementation. Inkjet-based bioprinting offers rapid fabrication speeds and high-resolution patterning capabilities, though material viscosity constraints and geometric complexity limitations persist.

Microalgae-based 3D bioprinting represents an interdisciplinary field combining microbiology, advanced materials science, and additive manufacturing. This technology enables precise spatial organization of microalgae-material interactions while allowing material innovations to enhance biological functionality. However, the field remains in its developmental phase, facing three primary challenges: (1) optimization of bioinks balancing printability with cellular viability, (2) development of application-specific fabrication protocols, and (3) cost of 3D bioprinting.

One of greatest challenges in 3D bioprinting technology lies in bioink material constraints. The available range of printable biocompatible materials suitable for biomedical applications remains relatively limited. To advance microalgae-based relevant research, there is a pressing need for functional bioinks that can properly support microalgal growth and structural integrity post-printing. Currently, many existing biomaterials are not ideal for particular applications due to insufficient biocompatibility, poor printability, or inability to maintain long-term stability in physiological environments. Addressing these limitations requires focused research on developing novel biocompatible materials that are simultaneously printable, structurally stable, and conducive to cell viability. Furthermore, extensive research into optimizing material formulation parameters is essential to push the field forward.

The second key challenge lies in the development of application-specific fabrication protocols. The utility of printed structures largely depends on the printing methodology employed. Developing printing strategies distinct from conventional bioprinting approaches to address diverse application scenarios will be a critical focus for future research. Recent innovations in multi-material integration capabilities and adaptive precision control architectures have demonstrated potential pathways. Notably, Zhang et al. developed an integrated bioprinting platform combining spatiotemporal microenvironment modulation with high-fidelity cellular deposition. This advancement exhibits significant potential for overcoming longstanding challenges in vascular network engineering and heterologous tissue fabrication, thereby establishing foundational technologies for next-generation organ manufacturing [117].

The third key challenge lies in the cost of bioprinting. The global bioprinting technologies market should reach USD 5.0 billion by 2027 from USD 2.4 billion in 2022 at a compound annual growth rate of 16.2% for the forecast period of 2022 to 2027 [118]. Demand for bioprinting equipment continues to grow across both industrial and academic sectors. However, high costs remain a significant barrier, particularly for small-to-medium-sized laboratories and clinical facilities. For instance, current high-end professional bioprinters with advanced automation can exceed USD 1 million in price [119]. The prohibitively high costs have somewhat hindered the research and application of 3D bioprinting technology. Therefore, to further advance 3D bioprinting development in the future, it has become imperative to develop low-cost printing systems.

### 5.2. Microalgae-Based 3D Bioprinting and Synthetic Biology

Bioprinting technology has illustrated a promising research paradigm in microalgae applications, positioning these photosynthetic organisms as pivotal components in synthetic biology frameworks. Amid escalating global crises including resource depletion, ecological degradation, and climate disruption, synthetic biological innovations emerge as promising solutions for reconciling socioeconomic development with ecological sustainability. Current implementations already demonstrate proven success across diverse domains such as medical therapeutics, renewable energy systems, advanced biomaterials, and sustainable agriculture, effectively challenging conventional production methodologies. The strategic convergence of microalgae biotechnology with 3D bioprinting platforms significantly amplifies functional potential of microalgae in bio-architectural design, photobioelectrochemical systems, regenerative medicine, and environmental remediation. This synergy fundamentally aligns with synthetic biology’s “bottom-up” engineering paradigm, which employs systematic Design–Build–Test–Learn (DBTL) cycles to construct programmable biological systems. Microalgae-based bioprinting operationalizes this framework through precise spatial organization of cellular components, enabling the exploration of next-generation materials with paradigm-shifting capabilities. Scientifically, 3D-bioprinted MB-ELMs exhibit inherent adaptive, self-regenerative, and stimulus-responsive characteristics that substantially surpass the performance thresholds of conventional synthetic substrates. The core scientific challenge centers on achieving precise spatiotemporal control over multicellular microenvironments while concurrently orchestrating material macrostructure evolution and dynamic functional modulation.

#### 5.2.1. Printing Microalgal Biomes/Communities

Synthetic biology enables the systematic selection or engineering of microbial chassis with tailored functionalities for 3D bioprinting applications. These bioengineered microorganisms are strategically assembled within three-dimensional biocompatible scaffolds that sustain cell viability and metabolic activity, optimizing cellular functionality to create specialized biosystems. Lee et al. engineered photosynthetic leaf-like architectures by immobilizing *Chlorella*-laden carboxymethyl cellulose/alginate hydrogels within 3D-printed matrices [120]. Joshi et al. developed an *Anabaena*-based biomimetic mushroom structure [85]. Three-dimensional bioprinting further serves as a prospective platform for elucidating microbial interaction dynamics. Microbial consortia exhibit complex ecological relationships spanning mutualism, competition, and predation, complemented by horizontal gene transfer mechanisms that enhance environmental adaptability. By enabling precise spatial patterning of microbial populations, 3D bioprinting facilitates the construction of particular community models, permitting systematic quantitative analysis of interaction mechanisms. Pioneering studies have already leveraged this technology to replicate coral-microalgae symbiotic systems [66,67], demonstrating how spatial configuration governs microbial cooperation and competition. This methodology provides potential resolution in studying how microenvironmental architecture influences phenotypic expression in microbial networks. Future research priorities will focus on developing sophisticated multicellular co-culture platforms to decode interspecies communication mechanisms, advancing both fundamental microbial ecology and applied synthetic biology.

#### 5.2.2. Advancing Microalgae-Based 4D Bioprinting

The integration of bioprinting with synthetic biology is poised to catalyze the emergence of 4D bioprinting, an evolutionary leap from static 3D-printed constructs to dynamic biological systems. Unlike conventional 3D printing, 4D bioprinting employs stimulus-responsive biomaterials that enable programmable shape morphogenesis or functional adaptation under specific environmental triggers [121]. Current research on 4D bioprinting remain in the exploratory phase [122,123], with no existing reports on microalgae-based 4D bioprinting thus far. For microalgae-based systems, this technology aims to engineer living materials with autonomous environmental perception and response capabilities, achieved through synergistic design of genetically engineered cells and spatially optimized architectures. Future investigations may prioritize three strategic directions:

(1) Intelligent Microalgal Response Systems: Genetic engineering of microalgae could yield chassis strains with tunable environmental sensitivity (e.g., light, humidity, ionic gradients). Coupled with 3D-printed hydrogel matrices, these strains may form adaptive bioreactors capable of structural reconfiguration or gene expression modulation in response to external cues. Such systems could advance applications in precision bioremediation, photosynthetic bioelectronics, and programmable tissue scaffolds.
(2) Spatiotemporally Controlled Microbial Consortia: By leveraging 4D bioprinting to position microalgae as primary producers within synthetic microbial ecosystems, interspecies dynamics can be systematically investigated including metabolic cross-feeding, quorum sensing, and horizontal gene transfer. These engineered communities may serve as self-regulating bioreactors, dynamically adjusting the functional outputs (e.g., metabolite production, carbon sequestration) in response to environmental fluctuations.
(3) Functionalized Bioprinted Edibles: Safety-certified genetically modified microalgae could be embedded into 4D-printed food matrices to enable targeted physiological effects, such as gut microbiome modulation or immunonutritional enhancement. This application necessitates rigorous optimization of algal viability, nutrient retention, and post-ingestion functionality within gastrointestinal environments.

While microalgae-based 4D bioprinting represents a field with great potential, its development faces inherent interdisciplinary challenges. Progress demands synergistic integration of materials science, polymer chemistry, and microbial biotechnology, supported by advanced infrastructure for precision bioprinting and multi-omics characterization. Consequently, the maturation of this field will require sustained interdisciplinary collaboration and incremental technological breakthroughs.

## Figures and Tables

**Table 1:** Comparison of different types of common 3D bioprinting methods.

Method	Resolution	Speed	Cell Density	Cell Viability	Viscosity	References
Extrusion	Low	Low	High	Low (40~80%)	High	[57,58]
Light projection	High	Fast	Moderate (<10^8^/mL)	High (>85%)	Low	[59,60]
Inkjet	High	Fast	Low (<10^6^/mL)	High (>85%)	Low	[61,62]

## References

[1] Layani M., Wang X., Magdassi S. (2018). Novel materials for 3D printing by photopolymerization. Adv. Mater..

[2] Guillemot F., Mironov V., Nakamura M. (2010). Bioprinting is coming of age: Report from the International Conference on Bioprinting and Biofabrication in Bordeaux (3B’09). Biofabrication.

[3] Mironov V. (2005). The second international workshop on bioprinting, biopatterning and bioassembly. Expert Opin. Biol. Ther..

[4] Ali H.R., Collier P., Bayston R. (2024). A three-dimensional model of bacterial biofilms and its use in antimicrobial susceptibility testing. Microorganisms.

[5] Malda J., Visser J., Melchels F.P., Jüngst T., Hennink W.E., Dhert W.J.A., Groll J., Hutmacher D.W. (2013). 25th anniversary article: Engineering hydrogels for Biofabrication. Adv. Mater..

[6] Kačarević Ž.P., Rider P.M., Alkildani S., Retnasingh S., Smeets R., Jung O., Ivanišević Z., Barbeck M. (2018). An introduction to 3D bioprinting: Possibilities, challenges and future aspects. Materials.

[7] Wang J., Cui Z., Maniruzzaman M. (2023). Bioprinting: A focus on improving bioink printability and cell performance based on different process parameters. Int. J. Pharm..

[8] Schuurman W., Khristov V., Pot M.W., van Weeren P.R., Dhert W.J.A., Malda J. (2011). Bioprinting of hybrid tissue constructs with tailorable mechanical properties. Biofabrication.

[9] Flombaum P., Gallegos J.L., Gordillo R.A., Rincón J., Zabala L.L., Jiao N., Karl D.M., Li W.K.W., Lomas M.W., Veneziano D. (2013). Present and future global distributions of the marine cyanobacteria Prochlorococcus and Synechococcus. Proc. Natl. Acad. Sci. USA.

[10] Chisti Y. (2007). Biodiesel from microalgae. Biotechnol. Adv..

[11] Falkowski P.G. (1994). The role of phytoplankton photosynthesis in global biogeochemical cycles. Photosynth. Res..

[12] Cui J., Sun H., Chen R., Sun J., Mo G., Luan G., Lu X. (2023). Multiple routes toward engineering efficient cyanobacterial photosynthetic biomanufacturing technologies. Green Carbon.

[13] Guo L.P., Zhang Y., Li W.C. (2017). Sustainable microalgae for the simultaneous synthesis of carbon quantum dots for cellular imaging and porous carbon for CO_2_ capture. J. Colloid Interface Sci..

[14] Weibel D.B., Garstecki P., Ryan D., DiLuzio W.R., Mayer M., Seto J.E., Whitesides G.M. (2005). Microoxen: Microorganisms to move microscale loads. Proc. Natl. Acad. Sci. USA.

[15] Li W., Zhong D., Hua S., Du Z., Zhou M. (2020). Biomineralized biohybrid algae for tumor hypoxia modulation and cascade radio-photodynamic therapy. ACS Appl. Mater. Interfaces.

[16] Hendrijantini N., Sitalaksmi R.M., Ari M.D.A., Hidayat T.J., Putri P.A.N., Sukandar D. (2020). The expression of TNF-α, IL-1β, and IL-10 in the diabetes mellitus condition induced by the combination of Spirulina and chitosan. Bali Med. J..

[17] Kalossaka L.M., Sena G., Barter L.M.C., Myant C. (2021). Review: 3D printing hydrogels for the fabrication of soilless cultivation substrates. Appl. Mater. Today.

[18] Xu W., Zhang X., Yang P., Långvik O., Wang X., Zhang Y., Cheng F., Österberg M., Willför S., Xu C. (2019). Surface engineered biomimetic inks based on UV cross-linkable wood biopolymers for 3D printing. ACS Appl. Mater. Interfaces.

[19] Markstedt K., Mantas A., Tournier I., Ávila H.M., Hägg D., Gatenholm P. (2015). 3D bioprinting human chondrocytes with nanocellulose-alginate bioink for cartilage tissue engineering applications. Biomacromolecules.

[20] Srubar W.V. (2021). Engineered living materials: Taxonomies and emerging trends. Trends Biotechnol..

[21] Nguyen P.Q., Courchesne N.M.D., Duraj-Thatte A., Praveschotinunt P., Joshi N.S. (2018). Engineered living materials: Prospects and challenges for using biological systems to direct the assembly of smart materials. Adv. Mater..

[22] Huang J., Liu S., Zhang C., Wang X., Pu J., Ba F., Xue S., Ye H., Zhao T., Li K. (2019). Programmable and printable Bacillus subtilis biofilms as engineered living materials. Nat. Chem. Biol..

[23] González-Camejo J., Viruela A., Ruano M.V., Barat R., Seco A., Ferrer J. (2019). Effect of light intensity, light duration and photoperiods in the performance of an outdoor photobioreactor for urban wastewater treatment. Algal Res..

[24] Ooms M.D., Dinh C.T., Sargent E.H., Sinton D. (2016). Photon management for augmented photosynthesis. Nat. Commun..

[25] Daly A.C., Prendergast M.E., Hughes A.J., Burdick J.A. (2021). Bioprinting for the biologist. Cell.

[26] Zhang Y.S., Haghiashtiani G., Hübscher T., Kelly D.J., Lee J.M., Lutolf M., McAlpine M.C., Yeong W.Y., Zenobi-Wong M., Malda J. (2021). 3D extrusion bioprinting. Nat. Rev. Methods Primers.

[27] Kumar V., Vlaskin M.S., Grigorenko A.V. (2021). 3D bioprinting to fabricate living microalgal materials. Trends Biotechnol..

[28] Lode A., Krujatz F., Brüggemeier S., Quade M., Schütz K., Knaack S., Weber J., Bley T., Gelinsky M. (2015). Green bioprinting: Fabrication of photosynthetic algae-laden hydrogel scaffolds for biotechnological and medical applications. Eng. Life Sci..

[29] Krujatz F., Lode A., Brüggemeier S., Schütz K., Kramer J., Bley T., Gelinsky M., Weber J. (2015). Green bioprinting: Viability and growth analysis of microalgae immobilized in 3D-plotted hydrogels versus suspension cultures. Eng. Life Sci..

[30] Windisch J., Reinhardt O., Duin S., Schütz K., Rodriguez N.J.N., Liu S., Lode A., Gelinsky M. (2023). Bioinks for space missions: The influence of long-term storage of alginate-methylcellulose-based bioinks on printability as well as cell viability and function. Adv. Healthc. Mater..

[31] Alasibi S., Kazir M., Israel Á., Livney Y.D. (2024). Algal protein-based 3D-printed fish-analogs as a new approach for sustainable seafood. Curr. Res. Food Sci..

[32] Fredricks J.L., Iyer H., McDonald R., Hsu J., Jimenez A.M., Roumeli E. (2021). Spirulina-based composites for 3D-printing. J. Polym. Sci..

[33] Xia X., Xu X., Lin C., Yang Y., Zeng L., Zheng Y., Wu X., Li W., Xiao L., Qian Q. (2020). Microalgal-immobilized biocomposite scaffold fabricated by fused deposition modeling 3D printing technology for dyes removal. ES Mater. Manuf..

[34] Kwak C., Ryu S.Y., Park H., Lim S., Yang J., Kim J., Kim J.H., Lee J. (2021). A Pickering emulsion stabilized by Chlorella microalgae as an eco-friendly extrusion-based 3D printing ink processable under ambient conditions. J. Colloid Interface Sci..

[35] Wang M., Lu X., Zheng X., Li W., Wang L., Qian Y., Zeng M. (2023). Rheological and physicochemical properties of Spirulina platensis residues-based inks for extrusion 3D food printing. Food Res. Int..

[36] Uribe-Wandurraga Z.N., Igual M., Reino-Moyón J., García-Segovia P., Martínez-Monzó J. (2021). Effect of microalgae (Arthrospira platensis and Chlorella vulgaris) addition on 3D printed cookies. Food Biophys..

[37] Uribe-Wandurraga Z.N., Zhang L., Noort M.W.J., Schutyser M.A.I., García-Segovia P., Martínez-Monzó J. (2020). Printability and physicochemical properties of microalgae-enriched 3D-printed snacks. Food Bioprocess Technol..

[38] Vieira M.V., Oliveira S.M., Amado I.R., Fasolin L.H., Vicente A.A., Pastrana L.M., Fuciños P. (2020). 3D printed functional cookies fortified with Arthrospira platensis: Evaluation of its antioxidant potential and physical-chemical characterization. Food Hydrocoll..

[39] Malik S., Hagopian J., Mohite S., Lintong C., Stoffels L., Giannakopoulos S., Beckett R., Leung C., Ruiz J., Cruz M. (2020). Robotic extrusion of algae-laden hydrogels for large-scale applications. Glob. Chall..

[40] Munaz A., Vadivelu R.K., St John J., Barton M., Kamble H., Nguyen N.T. (2016). Three-dimensional printing of biological matters. J. Sci. Adv. Mater. Devices.

[41] Balasubramanian S., Yu K., Meyer A.S., Karana E., Aubin-Tam M.E. (2021). Bioprinting of regenerative photosynthetic living materials. Adv. Funct. Mater..

[42] Léon R., Galván F. (1995). Glycerol photoproduction by free and Ca-alginate entrapped cells of Chlamydomonas reinhardtii. J. Biotechnol..

[43] Ramachandra Rao S., Tripathi U., Ravishankar G.A. (1999). Biotransformation of codeine to morphine in freely suspended cells and immobilized cultures of Spirulina platensis. World J. Microbiol. Biotechnol..

[44] Aguilar-May B., Sánchez-Saavedra M.P., Lizardi J., Voltolina D. (2007). Growth of *Synechococcus* sp. immobilized in chitosan with different times of contact with NaOH. J. Appl. Phycol..

[45] Stowers R.S., Allen S.C., Suggs L.J. (2015). Dynamic phototuning of 3D hydrogel stiffness. Proc. Natl. Acad. Sci. USA.

[46] Tóth G.S., Backman O., Siivola T., Xu W., Kosourov S., Siitonen V., Xu C., Allahverdiyeva Y. (2024). Employing photocurable biopolymers to engineer photosynthetic 3D-printed living materials for production of chemicals. Green Chem..

[47] Dranseike D., Cui Y., Ling A.S., Donat F., Bernhard S., Bernero M., Areeckal A., Qin X.H., Oakey J.S., Dillenburger B. (2025). Dual carbon sequestration with photosynthetic living materials. Nat Commun..

[48] Williams C.G., Malik A.N., Kim T.K., Manson P.N., Elisseeff J.H. (2005). Variable cytocompatibility of six cell lines with photoinitiators used for polymerizing hydrogels and cell encapsulation. Biomaterials.

[49] Vazquez-Martel C., Martins L.F., Genthner E., Almeida C., Quintana A.M., Bastmeyer M., Gómez Pinchetti J.L., Blasco E. (2024). Printing green: Microalgae-based materials for 3D printing with light. Adv. Mater..

[50] Pozzobon V., Otaola F., Arnoudts C., Lagirarde J. (2023). Impact of 3D printing materials on microalga Chlorella vulgaris. Bioresour. Technol..

[51] Zhao S., Guo C., Kumarasena A., Omenetto F.G., Kaplan D.L. (2019). 3D printing of functional microalgal silk structures for environmental applications. ACS Biomater. Sci. Eng..

[52] He F., Ou Y., Liu J., Huang Q., Tang B., Xin F., Zhang J., Jiang M., Chen S., Yu Z. (2022). 3D printed biocatalytic living materials with dual-network reinforced bioinks. Small.

[53] Wangpraseurt D., You S., Sun Y., Chen S. (2022). Biomimetic 3D living materials powered by microorganisms. Trends Biotechnol..

[54] Hinton T.J., Jallerat Q., Palchesko R.N., Park J.H., Grodzicki M.S., Shue H.J., Ramadan M.H., Hudson A.R., Feinberg A.W. (2015). Three-dimensional printing of complex biological structures by freeform reversible embedding of suspended hydrogels. Sci. Adv..

[55] Bernal P.N., Delrot P., Loterie D., Li Y., Malda J., Moser C., Levato R. (2019). Volumetric bioprinting of complex living-tissue constructs within seconds. Adv. Mater..

[56] Christensen K., Xu C., Chai W., Zhang Z., Fu J., Huang Y. (2015). Freeform inkjet printing of cellular structures with bifurcations. Biotechnol. Bioeng..

[57] Merceron T.K., Burt M., Seol Y.J., Kang H.W., Lee S.J., Yoo J.J., Atala A. (2015). A 3D bioprinted complex structure for engineering the muscle-tendon unit. Biofabrication.

[58] Deo K.A., Singh K.A., Peak C.W., Alge D.L., Gaharwar A.K. (2020). Bioprinting 101: Design, fabrication, and evaluation of cell-laden 3D bioprinted scaffolds. Tissue Eng. Part. A..

[59] Lim K.S., Levato R., Costa P.F., Castilho M.D., Alcala-Orozco C.R., van Dorenmalen K.M.A., Melchels F.P.W., Gawlitta D., Hooper G.J., Malda J. (2018). Bio-resin for high resolution lithography-based biofabrication of complex cell-laden constructs. Biofabrication.

[60] Ng W.L., Lee J.M., Zhou M., Chen Y.W., Lee K.X.A., Yeong W.Y., Shen Y.F. (2020). Vat polymerization-based bioprinting-process, materials, applications and regulatory challenges. Biofabrication.

[61] Xu T., Binder K.W., Albanna M.Z., Dice D., Zhao W., Yoo J.J., Atala A. (2012). Hybrid printing of mechanically and biologically improved constructs for cartilage tissue engineering applications. Biofabrication.

[62] Hong S., Song S.J., Lee J.Y., Jang H., Choi J., Sun K., Park Y. (2013). Cellular behavior in micropatterned hydrogels by bioprinting system depended on the cell types and cellular interaction. J. Biosci. Bioeng..

[63] Mohan D., Khairullah N.F., How Y.P., Sajab M.S., Kaco H. (2020). 3D printed laminated CaCO3-nanocellulose films as controlled-release 5-fluorouracil. Polymers.

[64] Wang Q., Sun J., Yao Q., Ji C., Liu J., Zhu Q. (2018). 3D printing with cellulose materials. Cellulose.

[65] Oh J.J., Ammu S., Vriend V.D., Kieffer R., Kleiner F.H., Balasubramanian S., Karana E., Masania K., Aubin-Tam M.E. (2024). Growth, distribution, and photosynthesis of Chlamydomonas reinhardtii in 3D hydrogels. Adv. Mater..

[66] Wangpraseurt D., You S., Azam F., Jacucci G., Gaidarenko O., Hildebrand M., Kühl M., Smith A.G., Davey M.P., Smith A. (2020). Bionic 3D printed corals. Nat. Commun..

[67] Wangpraseurt D., Sun Y., You S., Chua S., Noel S.K., Willard H.F., Berry D.B., Clifford A.M., Plummer S., Xiang Y. (2022). Bioprinted living coral microenvironments mimicking coral-algal symbiosis. Adv. Funct. Mater..

[68] Gong Y., Bi Z., Bian X., Ge A., He J., Li W., Shao H., Chen G., Zhang X. (2020). Study on linear bio-structure print process based on alginate bio-ink in 3D bio-fabrication. Bio-Des. Manuf..

[69] Sawa M., Fantuzzi A., Bombelli P., Howe C.J., Hellgardt K., Nixon P.J. (2017). Electricity generation from digitally printed cyanobacteria. Nat. Commun..

[70] Krujatz F., Dani S., Windisch J., Emmermacher J., Hahn F., Mosshammer M., Murthy S., Steingröwer J., Walther T., Kühl M. (2022). Think outside the box: 3D bioprinting concepts for biotechnological applications—Recent developments and future perspectives. Biotechnol. Adv..

[71] Ma Z., Cheah W.Y., Ng I.S., Chang J.S., Zhao M., Show P.L. (2022). Microalgae-based biotechnological sequestration of carbon dioxide for net zero emissions. Trends Biotechnol..

[72] (2021). Concrete needs to lose its colossal carbon footprint. Nature.

[73] Qiu J., Artier J., Cook S., Srubar W.V., Cameron J.C., Hubler M.H. (2021). Engineering living building materials for enhanced bacterial viability and mechanical properties. iScience.

[74] Song Q., Jiao K., Tonggu L., Wang L.G., Zhang S.L., Yang Y.D., Zhang L., Bian J.H., Hao D.X., Wang C.Y. (2019). Contribution of biomimetic collagen-ligand interaction to intrafibrillar mineralization. Sci Adv..

[75] Goodchild-Michelman I.M., Church G.M., Schubert M.G., Tang T.C. (2023). Light and carbon: Synthetic biology toward new cyanobacteria-based living biomaterials. Mater. Today Bio.

[76] Heveran C.M., Williams S.L., Qiu J., Artier J., Hubler M.H., Cook S.M., Cameron J.C., Srubar W.V. (2020). Biomineralization and successive regeneration of engineered living building materials. Matter.

[77] Zhu T., Paulo C., Merroun M.L., Dittrich M. (2015). Potential application of biomineralization by Synechococcus PCC8806 for concrete restoration. Ecol. Eng..

[78] Reinhardt O., Ihmann S., Ahlhelm M., Gelinsky M. (2023). 3D bioprinting of mineralizing cyanobacteria as novel approach for the fabrication of living building materials. Front. Bioeng. Biotechnol..

[79] Blankenship R.E., Tiede D.M., Barber J., Brudvig G.W., Fleming G., Ghirardi M., Gunner M.R., Junge W., Kramer D.M., Melis A. (2011). Comparing photosynthetic and photovoltaic efficiencies and recognizing the potential for improvement. Science.

[80] McCormick A.J., Bombelli P., Bradley R.W., Thorne R., Wenzel T., Howe C.J. (2015). Biophotovoltaics: Oxygenic photosynthetic organisms in the world of bioelectrochemical systems. Energy Environ. Sci..

[81] Zhu H., Wang H., Zhang Y., Li Y. (2023). Biophotovoltaics: Recent advances and perspectives. Biotechnol. Adv..

[82] Wang H.Y., Bernarda A., Huang C.Y., Lee D.J., Chang J.S. (2011). Micro-sized microbial fuel cell: A mini-review. Bioresour. Technol..

[83] Rosenbaum M., He Z., Angenent L.T. (2010). Light energy to bioelectricity: Photosynthetic microbial fuel cells. Curr. Opin. Biotechnol..

[84] Chen X., Lawrence J.M., Wey L.T., Schertel L., Jing Q., Vignolini S., Howe C.J., Kar-Narayan S., Zhang J.Z. (2022). 3D-printed hierarchical pillar array electrodes for high-performance semi-artificial photosynthesis. Nat. Mater..

[85] Joshi S., Cook E., Mannoor M.S. (2018). Bacterial nanobionics via 3D printing. Nano Lett..

[86] Liu L., Gao Y., Lee S., Choi S. 3D bioprinting of cyanobacteria for solar-driven bioelectricity generation in resource-limited environments. Proceedings of the 2018 40th Annual International Conference of the IEEE Engineering in Medicine and Biology Society (EMBC).

[87] Brar A., Kumar M., Soni T., Vivekanand V., Pareek N. (2021). Insights into the genetic and metabolic engineering approaches to enhance the competence of microalgae as biofuel resource: A review. Bioresour. Technol..

[88] Cao K., Cui Y., Sun F., Zhang H., Fan J., Ge B., Cao Y., Wang X., Zhu X., Wei Z. (2023). Metabolic engineering and synthetic biology strategies for producing high-value natural pigments in microalgae. Biotechnol. Adv..

[89] Giraldo Calderón N.D., Díaz Bayona K.C., Atehortúa Garcés L. (2018). Immobilization of the green microalga Botryococcus braunii in polyester wadding: Effect on biomass, fatty acids, and exopolysaccharide production. Biocatal. Agric. Biotechnol..

[90] Moreno-Garrido I. (2008). Microalgae immobilization: Current techniques and uses. Bioresour. Technol..

[91] Dawiec-Liśniewska A., Podstawczyk D., Bastrzyk A., Czuba K., Pacyna-Iwanicka K., Okoro O.V., Shavandi A. (2022). New trends in biotechnological applications of photosynthetic microorganisms. Biotechnol. Adv..

[92] Caldwell G.S., In-na P., Hart R., Sharp E., Stefanova A., Pickersgill M., Walker M., Unthank M., Perry J., Lee J.G.M. (2021). Immobilising microalgae and cyanobacteria as biocomposites: New opportunities to intensify algae biotechnology and bioprocessing. Energies.

[93] Ata A., Nalcaci O.O., Ovez B. (2012). Macro algae Gracilaria verrucosa as a biosorbent: A study of sorption mechanisms. Algal Res..

[94] Pagnanelli F., Jbari N., Trabucco F., Martínez M.E., Sánchez S., Toro L. (2013). Biosorption-mediated reduction of Cr(VI) using heterotrophically-grown Chlorella vulgaris: Active sites and ionic strength effect. Chem. Eng. J..

[95] Kariyawasam T., Petkovich M., Vriens B. (2024). Diclofenac degradation by immobilized Chlamydomonas reinhardtii and Scenedesmus obliquus. MicrobiologyOpen.

[96] Jiang M., Zheng J., Tang Y., Liu H., Yao Y., Zhou J., Lin W., Ma Y., Liu J., Zhou J. (2025). Retrievable hydrogel networks with confined microalgae for efficient antibiotic degradation and enhanced stress tolerance. Nat. Commun..

[97] Ou Y., Cao S., Zhang Y., Zhu H., Guo C., Yan W., Xin F., Dong W., Zhang Y., Narita M. (2023). Bioprinting microporous functional living materials from protein-based core-shell microgels. Nat. Commun..

[98] Sun Z., Wen H., Di Z., Zhang Y., Zhang S., Zhang Z., Zhang J., Yu Z. (2023). Photosynthetic living fiber fabrication from algal-bacterial consortia with controlled spatial distribution. ACS Biomater. Sci. Eng..

[99] Datta D., Weiss E.L., Wangpraseurt D., Hild E., Chen S., Golden J.W., Golden S.S., Pokorski J.K. (2023). Phenotypically complex living materials containing engineered cyanobacteria. Nat. Commun..

[100] Rademakers T., Horvath J.M., Blitterswijk C.A., LaPointe V.L.S. (2019). Oxygen and nutrient delivery in tissue engineering: Approaches to graft vascularization. J. Tissue Eng. Regen. Med..

[101] Centeno-Cerdas C., Jarquín-Cordero M., Chávez M.N., Hopfner U., Holmes C., Schmauss D., Machens H.G., Nickelsen J., Egaña J.T. (2018). Development of photosynthetic sutures for the local delivery of oxygen and recombinant growth factors in wounds. Acta Biomater..

[102] Qiao Y., Yang F., Xie T., Du Z., Zhong D., Qi Y., Li Y., Li W., Lu Z., Rao J. (2020). Engineered algae: A novel oxygen-generating system for effective treatment of hypoxic cancer. Sci Adv..

[103] Cohen J.E., Goldstone A.B., Paulsen M.J., Shudo Y., Steele A.N., Edwards B.B., Patel J.B., MacArthur J.W., Hopkins M.S., Burnett C.E. (2017). An innovative biologic system for photon-powered myocardium in the ischemic heart. Sci. Adv..

[104] Chen H., Cheng Y., Tian J., Yang P., Zhang X., Chen Y., Hu Y., Wu J. (2020). Dissolved oxygen from microalgae-gel patch promotes chronic wound healing in diabetes. Sci Adv..

[105] Dani S., Windisch J., Guerrero X.M.V., Bernhardt A., Gelinsky M., Krujatz F., Lode A. (2022). Selection of a suitable photosynthetically active microalgae strain for the co-cultivation with mammalian cells. Front. Bioeng. Biotechnol..

[106] Maharjan S., Alva J., Cámara C., Rubio A.G., Hernández D., Delavaux C., Correa E., Romo M.D., Bonilla D., Santiago M.L. (2021). Symbiotic photosynthetic oxygenation within 3D-bioprinted vascularized tissues. Matter.

[107] Wang X., Yang C., Yu Y., Zhao Y. (2022). In situ 3D bioprinting living photosynthetic scaffolds for autotrophic wound healing. Research.

[108] Liu H., Mei H., Jiang H., Jiang L., Lin K., Jiang M., Ding N., Li X., Gao Z., Liu B. (2025). Bioprinted symbiotic dressings: A lichen-inspired approach to diabetic wound healing with enhanced bioactivity and structural integrity. Small.

[109] Kolesky D.B., Homan K.A., Skylar-Scott M.A., Lewis J.A. (2016). Three-dimensional bioprinting of thick vascularized tissues. Proc. Natl. Acad. Sci. USA.

[110] Haraguchi Y., Kagawa Y., Sakaguchi K., Matsuura K., Shimizu T., Okano T. (2017). Thicker three-dimensional tissue from a “symbiotic recycling system” combining mammalian cells and algae. Sci. Rep..

[111] Trampe E., Koren K., Akkineni A.R., Senwitz C., Krujatz F., Lode A., Gelinsky M., Kühl M. (2018). Functionalized bioink with optical sensor nanoparticles for O~2~ imaging in 3D-bioprinted constructs. Adv. Funct. Mater..

[112] Fu Y., Chen T., Chen S.H.Y., Liu B., Sun P., Sun H., Chen F. (2021). The potentials and challenges of using microalgae as an ingredient to produce meat analogues. Trends Food Sci. Technol..

[113] Ferrazzano G.F., Papa C., Pollio A., Ingenito A., Sangianantoni G., Cantile T. (2020). Cyanobacteria and microalgae as sources of functional foods to improve human general and oral health. Molecules.

[114] Batista A.P., Gouveia L., Bandarra N.M., Franco J.M., Raymundo A. (2013). Comparison of microalgal biomass profiles as novel functional ingredient for food products. Algal Res..

[115] Mantihal S., Kobun R., Lee B.B. (2020). 3D food printing of as the new way of preparing food: A review. Int. J. Gastron. Food Sci..

[116] Liu H., Yu S., Liu B., Xiang S., Jiang M., Yang F., Tan W., Zhou J., Xiao M., Li X. (2024). Space-efficient 3D microalgae farming with optimized resource utilization for regenerative food. Adv. Mater..

[117] Zhang Z., Wu C., Dai C., Shi Q., Fang G., Xie D., Zhao X., Liu Y.J., Wang C.C.L., Wang X.J. (2022). A multi-axis robot-based bioprinting system supporting natural cell function preservation and cardiac tissue fabrication. Bioact. Mater..

[118] (2025). Global Bioprinting Market Research and Growth Forecast Analysis.

[119] Tong A., Pham Q.L., Abatemarco P., Mathew A., Gupta D., Iyer S., Voronov R. (2021). Review of low-cost 3D bioprinters: State of the market and observed future trends. SLAS Technol..

[120] Lee H., Shin D., Choi J., Ki C.S., Hyun J. (2022). Mimicry of the plant leaf with a living hydrogel sheet of cellulose nanofibers. Carbohydr. Polym..

[121] Taylor S., Mueller E., Jones L.R., Makela A.V., Ashammakhi N. (2024). Translational aspects of 3D and 4D printing and bioprinting. Adv. Healthc. Mater..

[122] Zhang H., Hua S., He C., Yin M., Qin J., Liu H., Zhou H., Wu S., Yu X., Jiang H. (2025). Application of 4D-printed magnetoresponsive FOGS hydrogel scaffolds in auricular cartilage regeneration. Adv. Healthc. Mater..

[123] Ding A., Lee S.J., Tang R., Gasvoda K.L., He F., Alsberg E. (2022). 4D cell-condensate bioprinting. Small.

